# Microscopic Properties of Hydrogen Peroxide Activated Crumb Rubber and Its Influence on the Rheological Properties of Crumb Rubber Modified Asphalt

**DOI:** 10.3390/ma12091434

**Published:** 2019-05-02

**Authors:** Bo Li, Hao Li, Yongzheng Wei, Xingjun Zhang, Dingbang Wei, Jia Li

**Affiliations:** 1 National and Provincial Joint Engineering Laboratory of Road & Bridge Disaster Prevention and Control, Lanzhou Jiaotong University, Lanzhou 730070, China; 13150017580@163.com (H.L.); vivibro@foxmail.com (Y.W.); weidingbang@163.com (D.W.); lijiageotech@hotmail.com (J.L.); 2 Key Laboratory of Highway Net Monitoring in Gansu Province, Gansu Hengda Road and Bridge Group Co. Ltd., Lanzhou 730070, China; zhangxingjun18@126.com; 3 Research and Development Center of Transport Industry of Technologies, Materials and Equipment of Highway Construction and Maintence, Gansu Road and Bridge Group Co. Ltd., Lanzhou 730050, China

**Keywords:** asphalt, crumb rubber, chemical activation, pore structure, surface morphology, viscoelasticity

## Abstract

Crumb rubber modified (CRM) asphalt binder has been affirmed to improve resistance to rutting, moisture susceptibility, low-temperature cracking, and asphalt durability. However, CRM has poor compatibility with asphalt since crumb rubber molecules are vulcanized. The objective of this study was to develop a new method to prepare activated crumb rubber using hydrogen peroxide (H_2_O_2_) solution and to explore the rheological properties of H_2_O_2_ activated CRM (ACRM) asphalt. Three different percentages of H_2_O_2_ solution were used to activate crumb rubber. The surface properties of oxidized rubber were analysed using scanning electron microscopy. Moreover, the pore structure in rubber powder was investigated. The rheological properties of bitumen samples obtained from treated and untreated rubber were characterized by conducting dynamic shear rheometer tests. The test results show that the average pore size of the crumb rubber after activation with H_2_O_2_ solution is significantly smaller than that of the inactivated crumb rubber, and the volume and surface area of the crumb rubber pores change with H_2_O_2_ solution activation in a certain pattern. With the increase in H_2_O_2_ solution content, the contact surface between the particles increases, the floccules and pores of the powder increase, and the interface degree between the crumb rubber powder and the asphalt is strengthened. Solubility of the rubber hydrocarbon and the release ability of the carbon black particles from the crumb rubber in the asphalt binder increase, but the mechanical properties of the crumb rubber, including the strength, elasticity, and wear resistance, decrease. As a result, a reduction is observed in the elasticity, viscosity, high-temperature rutting resistance, and elasticity of the ACRM asphalt.

## 1. Introduction

With the development of the automotive industry, large amounts of waste tires are generated, leading to serious environmental issues due to difficulty of decomposition and its huge stockpile [1,2,3]. The main composition of tires includes rubber, carbon black, steel wire, sulfur compound, and synthetic fibers. They are used in the asphalt paving industry because rubber and fibers contribute to improved asphalt pavement performance, which has been demonstrated by engineering experience. Scrap tires are treated to remove the steel wire and are ground to the desired size to produce crumb rubber. The crumb rubber is then added to the hot asphalt to produce a rubber-modified binder that has improved rheological properties [4,5]. It has been shown that crumb rubber modified asphalt is a solution to the environmental problems of waste tires [6,7]. The crumb rubber asphalt has good aging resistance, durability, and high temperature, and shows good road performance. At the same time, it has attracted the extensive attention of researchers because of its good economic and environmental benefits.

The dry process and wet process are two general methods for preparing granular rubber modified asphalt (CRMA) in previous studies. During the drying process, CRM is mixed with an aggregate as a filler for the asphalt mixture, which makes the interaction between asphalt and crumb rubber modified (CMR) very limited during the drying process. On the contrary, in the wet process, asphalt is blended with CRM for several hours at high temperature, which has the advantage that the wet process can modify rubber asphalt well, and the stability of rubber asphalt mixture produced by wet process is better than that of the dry process [8,9,10,11]. Therefore, the wet process is the preferred method to produce CRM asphalt.

The improvement of CRM asphalt using the wet process mainly depends on the interfacial properties of crumb rubber particles and the interaction between asphalt components and particles. The influence of CRM on asphalt binder has received significant attention from many researchers. Many studies have confirmed that asphalt binders modified with crumb rubber have higher viscosity compared to that of virgin asphalt binder. In addition, CRM is beneficial in terms of rutting resistance, moisture susceptibility, and low temperature cracking of asphalt binder and asphalt mixture [12,13,14,15]. As a result, the final pavement performance of asphalt mixture prepared using CRM asphalt binder can increase the pavement service life, reduce reflection cracking, decrease traffic noise, reduce maintenance cost, decrease pollution, and is very important for protecting the environment.

However, CRM has poor compatibility with asphalt because waste rubber powder is a cross-linked material, which limits the partial performance of CRM asphalt. The final performance of asphalt binder with CRM binder is significantly influenced by the physical and chemical properties of CRM. Because crumb rubber molecules are vulcanized, the three-dimensional network is difficult to crack in asphalt, even by blending at high temperatures for a long period of time. This leads to incompatibility between CRM and asphalt, and the settlement of rubber particles at the bottom of the bulk asphalt phase, which further affects the performance of CRM asphalt [16,17].

Many attempts have been made to break the C–S or S–S bond in rubber chemical linking networks (devulcanization) and improve the compatibility of CRM asphalt. These methods include high-speed shear disintegration, pre-mixing with the matrix, microwave treatment, and plasticization. The mechanical method adds raw materials to high-speed instruments under certain conditions, which initiates a chain scission reaction causing a fine rubber powder. The method is widely used and effective. Shatanawi suggested that hot water activation of crumb rubber is helpful for improving the compatibility between crumb rubber and asphalt binder because light oil fractions present in the crumb rubber particles were removed in this procedure, thus decreasing the phase segregation occurring between CRM particles and asphalt binder [18]. A more reactive rubber surface was created by adding furfural to crumb rubber, which will affect the rheological properties of the CRM binder. Xu introduced that an activated crumb rubber method using an ultrasonic focusing apparatus could more significantly improve the compatibility between the activated crumb rubber and neat asphalt binder [19]. Recently, microwave irradiation has been used for CRM pre-treatment before blending with an asphalt binder to improve the properties of rubber-asphalt blends in China [20,21]. Many studies have confirmed that higher surface activity and improved compatibility with asphalt due to microwave radiation treatment cleft the surface vulcanization network of the CRM [22].

However, the physical treatment of rubber powder is mostly energy-intensive, and the process is complex, making it difficult to meet actual needs. Therefore, researchers in various countries are looking for a cheaper and simpler method. It is similar to the physical method of breaking the internal S–S bond of the rubber powder and improving the surface activity, which adds different types of chemical agents to the rubber powder. Kocevski presented a study on the application of bulk polymerization of acrylic acid without using any initiator on ground rubber tire to surface modification [23]. The study indicated that viscosity and rheological properties are changed to increase the surface area of the CRM particles and formation of the anhydride on the CRM surface by grafting acrylic acid onto the surface of the CRM. Yu [24] oxidized the crumb rubber by sodium hypochlorite, which was used to prepare rubber-modified bitumen. The study indicated that the C–O and O–C=O content on the surface of the crumb tire rubber increased significantly after oxidizing modification. Xue [25] oxidized crumb rubber by benzoyl peroxide, which indicated that the bonds of C–C and C–H on the surface of crumb rubber broke to form surface active groups such as C–O and C=O after oxidizing modification with benzoyl peroxide, and the surface activity of crumb tire rubber significantly increased.

In this study, we developed a new method to prepare the activated crumb rubber using hydrogen peroxide (H_2_O_2_) solution and selected the activated crumb rubber as an asphalt modifier. Thereafter, the virgin asphalt was modified with H_2_O_2_ activated crumb rubber. The pore structure and microscopic morphology of the crumb rubber have been investigated by gas absorption experiments and scanning electron microscopy (SEM) tests. Based on the performance of H_2_O_2_ activated CRM (ACRM), the viscosity and viscoelasticity of ACRM were analysed by rotational viscosity tests and dynamic shear rheological tests.

## 2. Materials and Experiments

### 2.1. Materials

The SK virgin asphalt binder with a penetration grade of 90 was used in this study, which was obtained from the SK Petroleum Asphalt Factory, Southern Korea. The basic asphalt rheology tests are displayed in Table 1, including penetration, softening point, ductility, which meet the requirements of Chinese ‘Technical Specifications for Highway Asphalt Pavement Construction’ (JTG F40-2004). The basic test is carried out according to the Chinese specifications called ‘Test Rules for Asphalt and Asphalt Mixture in Highway Engineering’ (JTG E20—2011).

The 425-μm crumb rubber that was produced by the normal temperature method and provided by Jiuquan Rongtai Rubber Products Co., Ltd. (Gansu, China) in this study. Its fundamental properties meet the requirements of ‘Technical Specifications for CRM asphalt and Mixture Design and Construction’. Physical and chemical properties determined by the tests are summarized in Table 2.

It is observed from Table 2 that the main material of the crumb rubber is vulcanized rubber, and the main component of vulcanized rubber is nonpolar rubber hydrocarbon. In addition, rubber hydrocarbons and carbon black make up 52% and 30% of the crumb rubber, respectively. Carbon black particles are used as a reinforcing agent in the vulcanized rubber network structure, which is essential for improving mechanical properties such as strength, elasticity, and wear resistance of the crumb rubber.

### 2.2. Preparation of ACRM Asphalt

The crumb rubber is activated with 30% hydrogen peroxide (H_2_O_2_) solution by the weight of crumb rubber. The dry crumb rubber and H_2_O_2_ solution are first mixed thoroughly and then stored at room temperature for 24 h. Thereafter, the activated crumb rubber is dried in an oven at 80 °C until it reaches a constant weight. The main principal of this process method is that the H_2_O_2_ solution can oxidize the crumb rubber surface and change the surface condition of the crumb rubber, thereby changing the mechanical properties of the CRM asphalt.

To prepare the ACRM asphalt, the virgin asphalt binder is first heated to the flowing state in an oven at 135 °C, and then 500 g based asphalt binder is poured into a 1000 mL glass beaker, which is placed in a constant temperature oil bath to rapidly increase the temperature of the based asphalt binder to 190 °C. When the temperature of the based asphalt binders reaches 140 °C, 100 g of the H_2_O_2_ solution activated crumb rubber that was preheated in an oven at 80 °C is slowly added into the beaker and mixed well. After the crumb rubber and asphalt are mixed and reacted for 60 min, the desired ACRM asphalt is obtained [26]. The ratio of solution to crumb rubber is 0:1, 0.5:1, 1:1, and 1.5:1, and the four ratios are numbered as 0#, 1#, 2#, and 3#, respectively. The ACRM asphalt sample number is consistent with the number of the crumb rubber used to prepare the ACRM asphalt.

### 2.3. Characterization of Microscopic Properties of Crumb Rubber

The pore structure parameters including pore size distribution, pore surface area, and pore volume of the crumb rubber were determined by conducting Standard Test Method for Precipitated Silica–Surface Area by Single Point B.E.T. Nitrogen Adsorption (Norcross, GA, USA) according to (ASTM D5604-96(2012). The microstructures of both the activated and inactivated crumb rubber were characterized using a JSM-5600LV scanning electron microscope (Akishima, Tokyo, Japan).

### 2.4. ACRM Asphalt Viscoelastic Test

According to the ‘Testing Procedures for Asphalt and Asphalt Mixtures for Highway Engineering’ (JTG E20-2011), the Brookfield DV-2T viscometer is used to determine the viscosity of CRM asphalt. The same sample is tested for three times at a temperature of 180 °C with the 27# rotor, and each ACRM asphalt sample mass is 12.5 g. According to the ‘Road Engineering Waste Crumb Rubber Modified Asphalt’ (JTT 798-2011), the viscosity test data is analysed, and finally the viscosity of the ACRM asphalt is determined at torque of 50% [27].

The dynamic viscoelastic analysis of ACRM asphalt was performed using AR1500ex dynamic shear rheometer (DSR) tests. The appropriate amount of ACRM asphalt was placed on a 25 mm diameter plate. The distance between the two parallel plates was adjusted to 1050 μm. The sample was subjected to temperature scanning in the range of 58–82 °C, whereby each step of 6 °C was processed during temperature scanning with the strain control mode of 12% strain and 10 rad/s angular frequency.

## 3. Results and Discussion

### 3.1. Microscopic Morphology of Crumb Rubber

**Figure 1:**
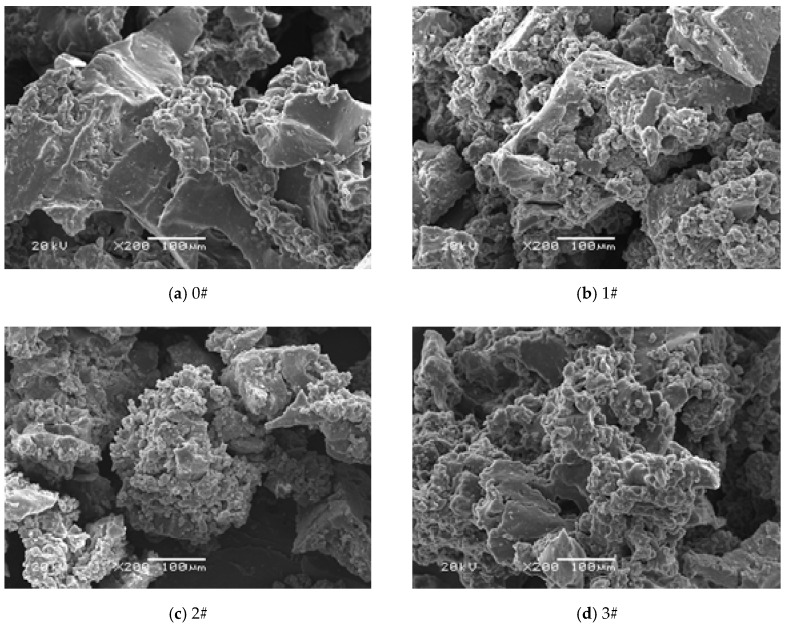
Microscopic morphology of crumb rubber activated by different proportions of H_2_O_2_.

Figure 1 shows the microscopic morphology of the crumb rubber activated with different H_2_O_2_ ratios. The 0# crumb rubber is found to be angular with a smooth surface, showing a certain flat shape. This structure is not conducive for the effective dispersion of the crumb rubber in the asphalt and does not adequately modify the asphalt [28,29]. After activation with H_2_O_2_ solution, the surface morphology of crumb rubber becomes rough, irregular, and uneven, exhibiting a fluffy and porous state. The surface layer is distinct, full of concave and convex areas, and is characterized by an increase in burrs and a flocculent structure. With the increase in the proportion of the H_2_O_2_ solution, the surface of the crumb rubber becomes rougher, and the texture changes from flocculent to a fluffier texture. This flocculent structure causes the contact area between crumb rubber and asphalt to increase correspondingly, which is conducive to the penetration of light oil in asphalt into the interior of crumb rubber. In this way, the modification effect of crumb rubber on asphalt can be highlighted, and the dissolution of crumb rubber in asphalt can be accelerated.

### 3.2. Pore Structure of Crumb Rubber Activated by H_2_O_2_

**Figure 2:**
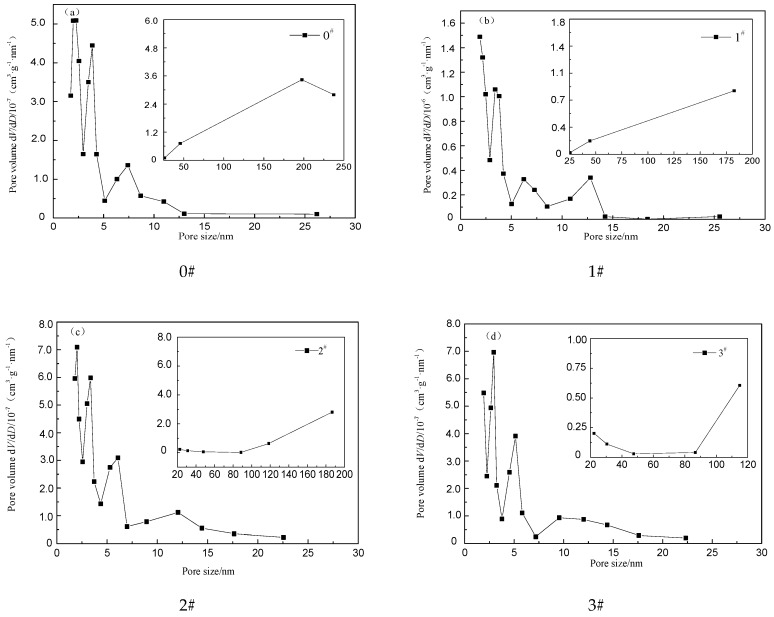
Pore distribution of crumb rubber activated by H_2_O_2._

Figure 2 shows pore size distribution of the crumb rubber with different percentages of H_2_O_2_ respectively. It can be seen that the pore size distribution of rubber powder is more complex, and the pore size distribution is mainly concentrated in the range of 1.73–237.40 nm, with a peak value in the range of pore sizes less than 15 nm. The first peak value of the pore volume appears at a pore size of approximately 2 nm for all four samples, and the second peak value appears at a pore size in the range of 3 to 4 nm. In the range of 5 to 15 nm, the pore size distribution curve of the 0# sample only shows a peak at approximately 7.5 nm, while the other three samples show two peaks but the peak values are relatively small. The pore size of 0# and 1# samples begin to increase when the pore size is larger than 25 nm, and the pore size of the 2# and 3# samples begin to increase when the pore size is larger than 80 nm, while the critical value of the pore size growth is greater than that of the 0# and 1# sample. All samples exhibit the largest peak values in the range of 2 to 4 nm over the entire pore size distribution range, indicating that the pore proportion of the crumb rubber is the largest in the pore size range of 2 to 4 nm.

**Figure 3:**
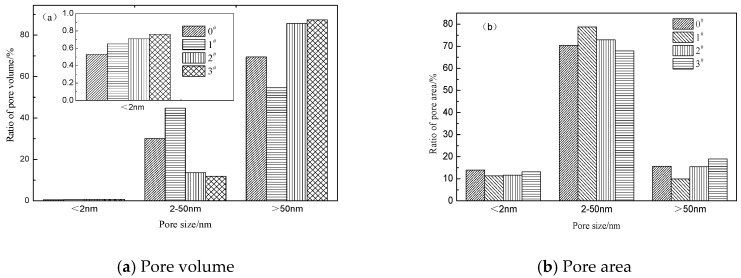
Pore volume and pore area distribution of crumb rubber activated by H_2_O_2_.

According to International Union of Pure and Applied Chemistry standards, materials can be classified as microporous materials (pore sizes less than 2 nm), mesoporous materials (pore sizes between 2 and 50 nm), and macroporous materials (pore sizes greater than 50 nm) [30]. Figure 3 shows the pore volume and pore surface area distribution of the crumb rubber with different mass ratios of H_2_O_2_ solution activation. Figure 3a shows that the macropore volume of each sample accounts for 54.60–87.37% of the total pore volume. For 1# to 3# samples, the micropore and macropore volumes increase gradually, and the mesoporous volume decreases gradually with increasing percentage of H_2_O_2_ solution. Compared with the 0# unmodified crumb rubber sample, when the mass ratio of H_2_O_2_ to crumb rubber is 0.5, the volumes of micropores and mesopores of the crumb rubber increase, and the volume of macropores decreases with increasing percentage of H_2_O_2_ solution. When the mass ratio of H_2_O_2_ to crumb rubber is 1.0 and 1.5, the pore and macropore volumes of the crumb rubber increase, and the mesopore volume decreases with increasing percentage of H_2_O_2_ solution. Figure 3b shows that the pore surface area is mainly concentrated in the mesoporous range, accounting for 67.84–78.72% of the total pore surface area. It can be observed that the mesopores account for the highest contribution to the pore area of the crumb rubber. For 1# to 3# samples, as the percentage of the H_2_O_2_ solution increases, the surface areas of the micropores and macropores increase, while the mesopore surface area decreases. Compared with 0# unmodified crumb rubber, when the mass ratio of H_2_O_2_ to crumb rubber is 0.5 and 1.0, the micropore and mesopore surface areas of the crumb rubber decrease, and the mesopore area increases with increasing percentage of H_2_O_2_ solution. When the mass ratio of H_2_O_2_ to crumb rubber is 1.5, the micropore and mesopore surface areas of the crumb rubber decrease, and the macropore surface area increases with increasing percentage of H_2_O_2_ solution.

**Figure 4:**
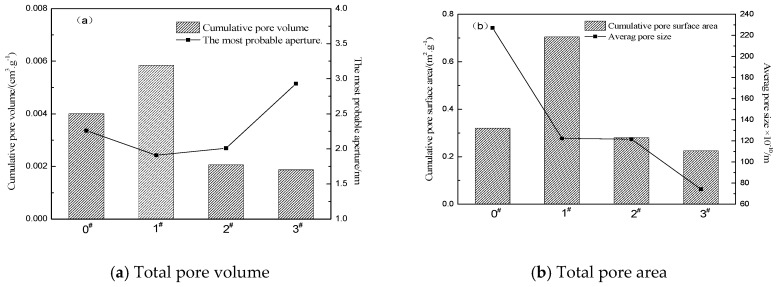
Total pore volume and total pore area of crumb rubber activated by H_2_O_2._

Figure 4 shows the cumulative pore volume and cumulative pore surface area of the crumb rubber after different ratios of H_2_O_2_ activation. It can be observed that the cumulative pore volume and cumulative pore surface area of the 1# crumb rubber sample are the largest. For the sample of 1# to 3#, with the increase in the percentage of H_2_O_2_ solution, the cumulative pore volume and cumulative pore area of the crumb rubber gradually decrease. Compared with the 0# unmodified crumb rubber, when the mass ratio of H_2_O_2_ is 0.5, the cumulative pore volume and cumulative pore surface area of the crumb rubber significantly increase. When the mass ratio of H_2_O_2_ is 1.0 and 1.5, the cumulative pore volume and cumulative pore surface area of the crumb rubber decrease. It can be observed from Figure 3a that the most probable pore size of the crumb rubber first decreases and then increases with increasing mass ratio of H_2_O_2_. When the mass ratio of H_2_O_2_ is 0.5, the most probable pore size of the crumb rubber is the smallest. When the mass ratio of H_2_O_2_ is 1.5, the most probable pore size of the crumb rubber is the largest. As depicted in Figure 3b, the 0# crumb rubber has a maximum pore size of 227.17 Å (1 Å = 1 × 10^−10^ m). With the increase in the H_2_O_2_ mass ratio, the pore size of the crumb rubber decreases gradually. When the mass ratio of H_2_O_2_ is 1.5, the crumb rubber has a minimum pore size of 74.14 Å. The pore size of the crumb rubber significantly decreases after being activated by H_2_O_2_.

### 3.3. ACRM Asphalt Viscoelastic

**Figure 5:**
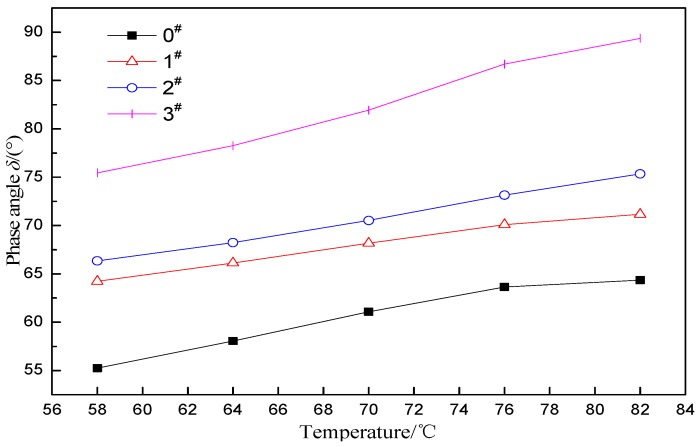
Phase angle of ACRM asphalt.

The phase angle δ represents the ratio of the viscous component to the elastic component in the asphalt. The larger δ indicates a smaller asphalt elasticity. Figure 5 shows the δ of the rubber asphalt prepared by different proportions of H_2_O_2_ activated crumb rubber. It can be seen that the δ increases gradually with the increase of temperature, indicating that the elasticity decreases. At the same temperature, δ increases with increase in H_2_O_2_ content, indicating that the crumb rubber activated with the H_2_O_2_ solution reduces the elasticity of the ACRM asphalt. A greater proportion of H_2_O_2_ results in a larger decrease in elasticity of ACRM asphalt. The minimum value of 3# asphalt is larger than the maximum value of the other three asphalts, indicating that it will significantly reduce the elasticity of the ACRM asphalt with H_2_O_2_ activated crumb rubber, with a mass ratio of 1.5.

**Figure 6:**
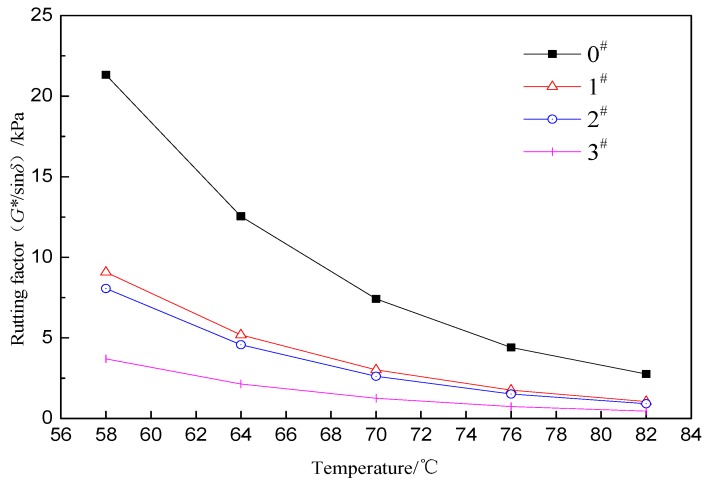
Rutting factor of ACRM asphalt.

Rutting factor G*/sinδ is used to measure the rutting resistance ability of asphalt under high temperature conditions. The larger the rutting factor is, the better the rutting resistance ability of the asphalt is under high temperature [31]. Figure 6 shows the rutting factor of ACRM asphalt prepared by different percentages of H_2_O_2_ activated crumb rubber. It can be seen that G*/sinδ decreases with increasing temperature suggesting that the rutting resistance ability of ACRM asphalt decreases during the heating process. Moreover, the 0# sample has the largest reduction, and the 3# has the smallest reduction. G*/sinδ declined significantly because most of the complex structure formed by the vulcanized rubber and carbon black in the crumb rubber gradually decomposes with the dissolution, and the nonpolar rubber hydrocarbons on the surface of the crumb rubber are dissolved in nonpolar components, such as aromatics and saturated fractions, that are adsorbed on the crumb rubber surface, based on the principle of similar compatibility [32]. Therefore, mechanical properties such as strength and elasticity of the crumb rubber are reduced.

This finding also reveals the full temperature dependence of the viscoelastic material of asphalt. Rutting factor G*/sinδ decreases rapidly with increasing temperature in the range of 58–76 °C. In other words, the high temperature deformation resistance of ACRM asphalt is rapidly reduced. In the range of 76–82 °C, G*/sinδ it decreases slowly. It shows that the high temperature deformation resistance of ACRM asphalts exhibits almost no change within the temperature range. In addition, with the increase in temperature, the difference of G*/sinδ at the same temperature gradually decreases, and the resistance to rutting is nearly uniform. Rutting factor G*/sinδ changes with the same pattern as the viscosity for all samples. With increasing H_2_O_2_ content, the G*/sinδ of ACRM asphalt decreases gradually, in which 1# is close to 2#, and 3# exhibits the smallest G*/sinδ.

**Figure 7:**
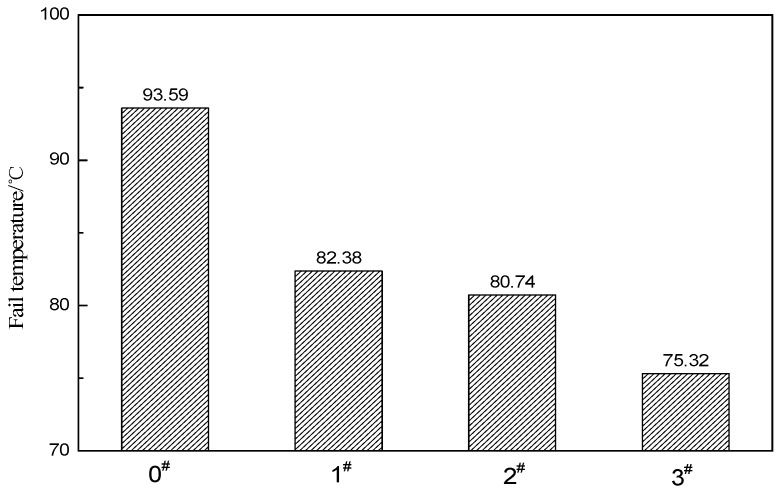
Failure temperature of ACRM asphalt.

The failure temperature, for which G*/sinδ less than 1.0 kPa, is often used to determine the performance grade of an asphalt [33]. The test results are shown in Figure 7, which illustrate that the ACRM asphalt failure temperatures are greater than 70 °C, and the 0# ACRM asphalt has the highest failure temperature, while the 3# ACRM asphalt has the lowest. Although the rutting resistance ability of the ACRM asphalt decreased after crumb rubber activated by H_2_O_2_, the failure temperature (the testing temperature when G*/sinδ is 1.0 kPa) of the 3# sample with the largest reduction still reached 75.32 °C. This shows that the ACRM asphalt prepared by the crumb rubber, activated by H_2_O_2_ solution, exhibits good high temperature resistance to rutting deformation.

**Figure 8:**
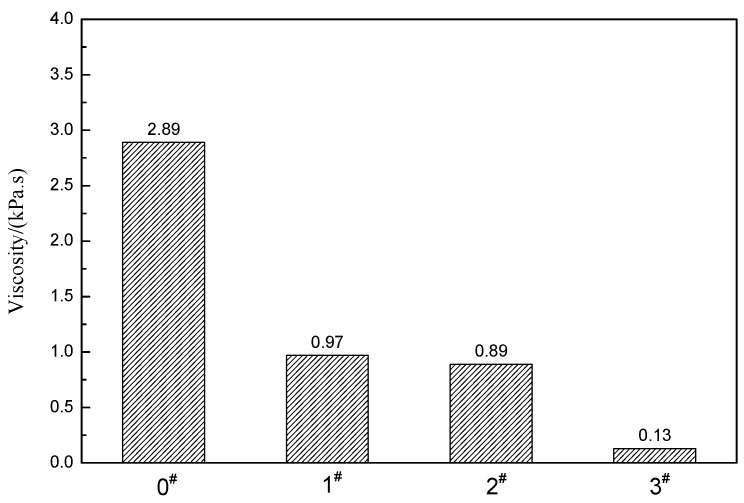
Viscosity of ACRM asphalt.

Figure 8 shows the viscosity of ACRM asphalt prepared by different mass ratios of H_2_O_2_ activated crumb rubber. The viscosity of ACRM asphalt gradually decreases with the increase in the H_2_O_2_ ratio. The viscosity of the ACRM asphalt prepared by the crumb rubber activated with H_2_O_2_ is reduced by 66.4%, 69.2%, and 95.5%, compared with the 0# ACRM asphalt. The viscosities of 1# and 2# are relatively close, and 3# sample shows the largest viscosity reduction. When the ratio of the H_2_O_2_ solution is changed from 0.5:1 to 1:1, the viscosity reduction of ACRM asphalt is limited. When the ratio of the H_2_O_2_ solution exceeds a certain range, the viscosity is significantly reduced. The lower viscosity of the ACRM asphalt is beneficial for pumping asphalt and mixing and paving the asphalt mixture, reducing the construction temperature, and providing technical support for the green construction of pavement.

### 3.4. Creep Recovery Performance of ACRM Asphalt

To better evaluate the recovery performance of rubber modified asphalt under loading [34], the creep recovery test is conducted, with a loading stress of 30 Pa, loading time of 1 s, unloading time of 9 s, and a testing temperature of 60 °C.

**Figure 9:**
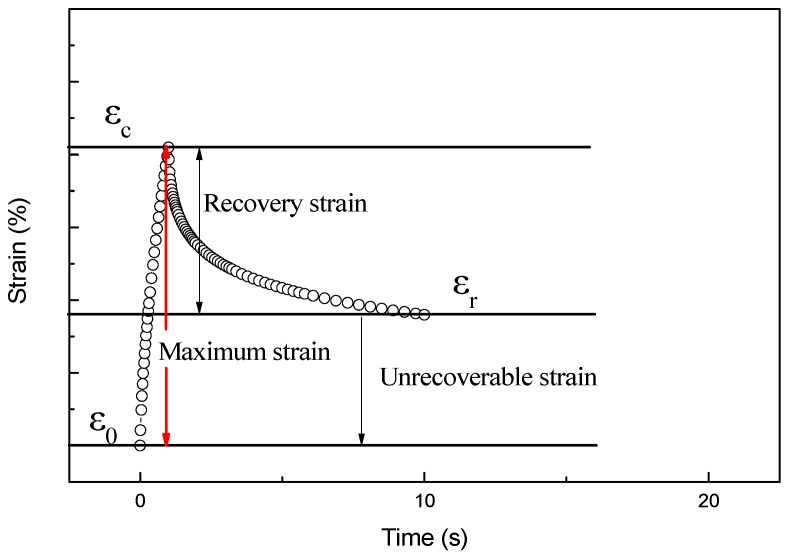
Scheme of creep recovery test.

As shown in Figure 9, the initial strain at which stress begins to load is ε_0_, and the peak strain is ε_c_, while the residual strain at the end of the unloading cycle is recorded as ε_r_. The strain recovery rate R is calculated using Equation (1).

(1) $$R\;=\;\frac{\varepsilon_{c}{-\varepsilon }_{r}}{\varepsilon_{c}{-\varepsilon }_{0}}\times 100\%$$

**Figure 10:**
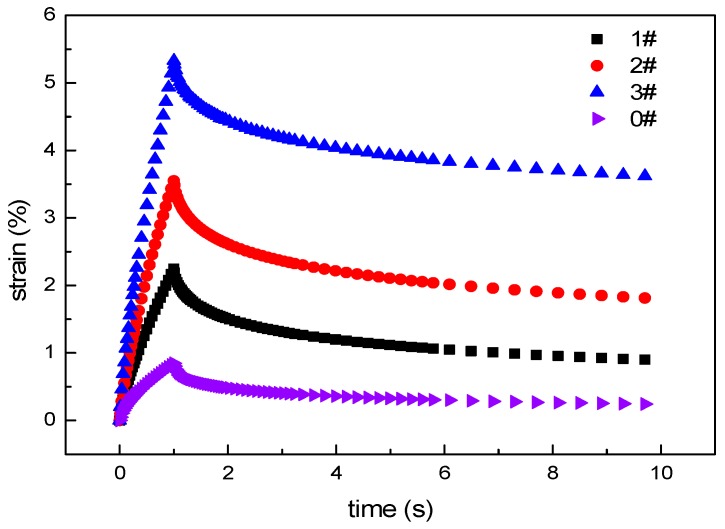
Results of creep recovery test.

The results of the creep recovery test are shown in Figure 10. During the stress loading process, the strain of the ACRM asphalt gradually increases with increase in time. The larger the mass ratio of the H_2_O_2_ solution is, the faster the strain of the ACRM asphalt increases, resulting in a larger peak strain. In the stress unloading phase, the strain of the ACRM asphalt gradually decreases with time.

**Figure 11:**
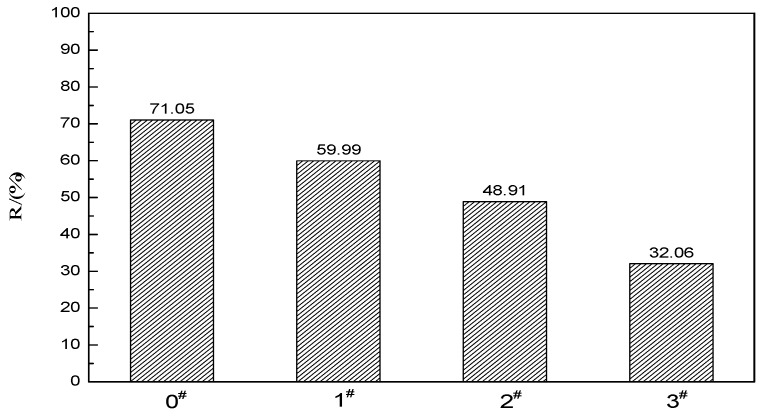
Strain recovery rate.

The strain recovery rate of ACRM asphalt is shown in Figure 11. After the crumb rubber is activated, the strain recovery rate of the ACRM asphalt decreases. The larger the proportion of H_2_O_2_ solution is, the smaller the strain recovery rate of ACRM asphalt is, indicating that the activation of the rubber powder by H_2_O_2_ solution reduces the elastic recovery ability of ACRM asphalt.

## 4. Conclusions

In this study, different proportions of hydrogen peroxide solution were used to prepare activated crumb rubber, and activated rubber asphalt was prepared by activated crumb rubber. The pore structure of activated crumb rubber and rheology of activated rubber asphalt were studied. Based on the test results in this study, the following conclusions were drawn from the materials used in this study.

(1) As the proportion of H_2_O_2_ solution increases, the average pore size of the crumb rubber gradually decreases from 227.17 Å to 74.14 Å, and the average pore size decreases significantly. The large pores of the crumb rubber account for 54.60–87.37% of the total pore volume, contributing the most to the cumulative pore volume, and the mesopores accounted for 67.84–78.72% of the total pore area, contributing the most to the cumulative pore area. When the mass ratio of H_2_O_2_ is 0.5, the most probable pore size of the crumb rubber is the smallest, while an H_2_O_2_ mass ratio of 1.5 corresponds to the largest most probable crumb rubber size.

(2) The viscosity, elasticity, and high temperature rutting resistance of rubber asphalt decrease after the crumb rubber is activated with H_2_O_2_ solution. Compared with unmodified ACRM asphalt, the viscosity of the H_2_O_2_ solution ACRM asphalt is reduced by 66.4%, 69.2%, and 95.5%, with increasing percentage of H_2_O_2_ solution at 0.5:1, 1:1, and 1.5:1, respectively. The activation of the H_2_O_2_ solution reduces the elastic recovery ability of rubber asphalt.

(3) After the crumb rubber is activated with H_2_O_2_ solution, the contact surface between the particles increases, and the surface flocks and pores gradually increase. As the proportion of H_2_O_2_ solution increases, the flock on the surface of the crumb rubber becomes fluffier, and the pores are denser. It can be inferred that the changes in the apparent morphology and pore structure of the crumb rubber are the main reasons that cause the change of the pore structure parameters of the crumb rubber.

(4) The gradual increase in the microscopic pores of the crumb rubber increases the adsorption of the crumb rubber for the nonpolar components in the asphalt, making the nonpolar rubber hydrocarbons of the crumb rubber more soluble in the nonpolar components of the asphalt. As the dissolution gradually occurs, the carbon black particles of the crumb rubber are gradually released and dispersed in the asphalt, reducing the mechanical properties of the crumb rubber, as well as the elasticity, viscosity, and anti-rutting ability of the CRM asphalt.

## Figures and Tables

**Table 1:** Properties of SK Asphalt Binder.

Test Properties		Specification	Result
Penetration (25 °C, 100 g, 5 s)/0.1 mm		80–100	92.0
Softening point/°C		≥42	46.2
Ductility (5 cm/min, 10 °C)/cm		≥100	>100
RTFOT (163 °C, 85 min)	Loss of quality/%	≤ ±0.8	0.07
	Penetration ratio/%	≥54	70
	Ductility (5 cm/min, 10 °C)/cm	≥6	9.0

**Table 2:** Physical and chemical properties of crumb rubber.

Test Properties	Bulk Density/(kg/m^3^)	Moisture Content/%	Metal Content/%	Fiber Content/%	Ash Content/%	Acetone Extract/%	Carbon Black Content/%	Rubber Hydrocarbon Content/%
Technical indexes	260–460	<1	<0.03	<1	≤8	≤22	≥28	≥42
Test results	302.5	0.0	0.009	0.065	7.3	7.2	30	52
